# Insomnia and common mental disorder among patients with pre-existing chronic non-communicable diseases in southern Ethiopia: a survey during COVID-19 pandemic

**DOI:** 10.3389/fpsyt.2023.1142926

**Published:** 2023-09-15

**Authors:** Mohammed Ayalew, Bedilu Deribe, Siraj Hussen, Semira Defar, Emnet Tesfaye, Abel Gedefaw

**Affiliations:** ^1^School of Nursing, College of Medicine and Health Sciences, Hawassa University, Hawassa, Ethiopia; ^2^School of Medical Laboratory, College of Medicine and Health Sciences, Hawassa University, Hawassa, Ethiopia; ^3^Department of Midwifery, College of Medicine and Health Sciences, Hawassa University, Hawassa, Ethiopia; ^4^Department of Emergency and Critical Care Medicine, School of Medicine, College of Medicine and Health Sciences, Hawassa University, Hawassa, Ethiopia; ^5^Department of Obstetrics and Gynecology, School of Medicine, College of Medicine and Health Sciences, Hawassa University, Hawassa, Ethiopia

**Keywords:** common mental disorder, insomnia, COVID-19, NCDs, Ethiopia

## Abstract

**Background:**

COVID-19 has been causing significant mental health problems and other health-related issues. Despite the fact that COVID-19 has a significant impact on chronic disease patients, there is scant research on insomnia, common mental health disorders (CMD), and their associated factors among chronic disease patients.

**Objective:**

The purpose of this study was to assess the prevalence of insomnia and common mental disorders (CMD) and their associated factors among patients with pre-existing chronic NCDs in Sidama, southern Ethiopia.

**Methods:**

A multicenter cross-sectional study was undertaken between June 1 and September 1, 2021. The study included 633 participants. CMD and insomnia were assessed using a 20-item Self-Reported Questionnaire (SRQ-20) and a 7—item Insomnia Severity Index (ISI) scale, respectively. To describe the various variables, descriptive statistics were used. We performed multivariable logistic regression analysis to identify independent factors associated with CMD and insomnia. A value of p < 0.05 was considered statistically significant at a 95% confidence interval.

**Results:**

The prevalence of insomnia and CMD was found to be 39.3% and 46.8%, respectively. Being merchant (AOR = 0.33; 95% CI = 0.13, 0.82), having a diagnosis of diabetes mellitus (AOR = 1.89; 95% CI = 1.04, 3.46), comorbid diagnosis (AOR = 3.96; 95% CI = 2.27, 6.89), low social support (poor (AOR = 3.37; 95% CI = 1.51, 7.57) and moderate (AOR = 3.13; 95% CI = 1.46, 6.69)), symptoms of insomnia (AOR = 12.08; 95% CI = 7.41, 19.72) and poor quality of life (QOL) (AOR = 1.67; 95% CI = 1.04, 2.72) were independent predictors of CMD. We also found out that, having cardiovascular disorders (CVDs) (AOR = 2.48; 95% CI = 1.18, 5.19), CMD (AOR = 12.09; 95% CI = 7.46, 19.61), and poor QOL (AOR = 2.04; 95% CI = 1.27, 3.26) were significantly associated with insomnia symptoms.

**Conclusion:**

Our study suggests that substantially high prevalence of CMD and insomnia. Significant association between CMD and occupation, diagnosis, comorbidity, social support, insomnia, and QOL were found. We also revealed that having CVDs, CMD, and poor QOL were significantly associated with insomnia symptoms. Therefore, dealing with the mental health problems of patients with chronic NCDs is an essential component of public health intervention during the COVID-19 pandemic.

## Introduction

Noncommunicable diseases (NCDs) are a group of chronic diseases that significantly increase morbidity and mortality in the community, including cardiovascular disease, stroke, cancer, diabetes, and mental health issues. NCDs have been exerting a significant global burden and considered as primary public health agenda for decades (1). NCDs account for three out of every 4 years lived with disability (YLD), whereas mental disorders contribute for nearly one-fourth of all YLD (2, 3). Mental illness and NCDs are commonly related by underlying individual, community, and social characteristics, and they commonly have a bidirectional relationship (4). Chronic NCDs, for instance, can lead to depression and anxiety, while mental disorders may result in decreased treatment seeking, poor adherence to treatment, and worse the outcome of NCD (5). Studies have discovered an association between chronic pain, long-term neurological conditions, or kidney illness, and depression, anxiety, and sleep problems (6–8).

COVID-19 also known as severe acute respiratory syndrome coronavirus-2 (SARS-CoV2), first appeared in the year 2020, causing a global health crisis and the collapse of several healthcare systems (9, 10). The ongoing COVID-19 outbreak overwhelmed the healthcare system, resulting in remarkable impact in the clinical management of patients with pre-existing chronic illness (11). Patients with pre-existing medical disorders such as diabetes mellitus (DM), hypertension, and cancers are considered to be among the most sensitive to COVID-19 infection, with higher severity and death (12). Patients with hypertension (6%), diabetes (7.3%) and cardiovascular disease (10.5%) had a higher case-fatality rate than the general population (2.3%) (13).

The COVID-19 pandemic is claimed to be the cause of a significant increase in the global prevalence of mental disorders such as sleep problems, anxiety and depression. In addition, WHO expressed concern regarding the influence of COVID-19 on an individual’s mental health and psychosocial implications (14). Evidences showed that individuals’ mental health were worsened during and after the COVID-19 pandemic, compared to before the pandemic (15, 16). Another study found that COVID-19 lockdown was linked to poor quality of sleep, sleep deprivation, and depressed symptoms (17). Moreover, a systematic review found that the COVID-19-affected population in many nations experienced somewhat high rates of insomnia, anxiety, depression, posttraumatic stress disorder, and psychological distress symptoms (18).

People may exhibit signs of depression, anxiety, and stress such loneliness, denial, insomnia, despair, boredom, and irritability, and they even face a greater chance of committing suicide (19). Isolation and loneliness, a lack of treatment options, and limited access to medical care, as well as widespread media coverage of COVID-19 infection’s high infectivity, mortality, and diseases predisposing to an adverse course, all contribute to a significant psychological burden that leads to distress and sleep disturbances (20, 21).

Insomnia is described as subjective complaints about difficulty of falling asleep, nocturnal awakenings, early morning awakening, or non-refreshing sleep (22). Insomnia is one of the most common sleep related disorder, affecting 5–19% of people globally (23, 24). According to Budhiraja and colleagues, one-fourth (27.3%) of people with chronic obstructive lung disease had sleep problems (25). One of the most prevalent issues during the pandemic is sleep disturbance. According to a recent systematic review and meta-analysis (SRMA) study, patients with COVID-19 (57%) had the highest pooled prevalence of insomnia, followed by healthcare professionals (31%), and the general population (18%) (26). The pooled prevalence of insomnia in the Chinese population was 39.1%, according to a recent systematic review (27). A similar review indicated that the prevalence of insomnia in Africa during the pandemic were found to be 28.1%, with higher prevalence in North Africa than Sub-Saharan Africa (31% *Vs* 24%) (28).

A study was conducted during the initial period of COVID-19 outbreak in China, and it indicated that 8.1% had moderate to severe level of stress, 16.5% had moderate to severe symptoms of depression, and 28.8% had moderate to severe symptoms of anxiety (29). A national survey found that distress was highly prevalent in the general population, with rates of 35, 60, and 45% in China, Iran, and the United States, respectively (30).

Furthermore, sleep disturbances were associated with higher levels of mental distress (26). Sleep is essential for both mental and physical well-being, and it can even enhance immunity and resistance to infections, not to mention the different metabolic, autonomic, and inflammatory systems that are impacted by sleep deprivation (31, 32). Insomnia is becoming more often considered as a distinct and comorbid disorder that requires sleep-focused therapy, despite the fact that sleep disturbance can be an indication of psychological problems (33). In addition to resulting in distress, insomnia initiates and/or worsens other mental health problems, contributes to stress and disability when associated with mental disorders, and frequently remains after other symptoms of mental disorders have subsided. This could be because lack of sleep sensitizes the stress system even more, resulting in more stressful impressions of life events as well as diminished resilience and stress recovery (34–39).

It has been established that the COVID-19 pandemic significantly affects the development of the illness and survival of NCD patients. As a result, worries regarding the mental health of NCD patients have increased. Comorbid anxiety and depression make it difficult for patients to take their medications, which decreases therapeutic safety and efficacy and raises the risk of disability and death. Comorbid mental problems, however, are frequently ignored and sometimes not adequately treated (40). Despite the fact that COVID-19 has a significant impact on chronic disease patients, there is scant research on insomnia, CMD, and their associated factors among chronic disease patients. We hypothesized that COVID-19 has increased the risk of developing insomnia and mental disorders in patients with pre-existing chronic medical illnesses. As a result, the purpose of this study was to examine the prevalence of insomnia and CMD of patients with pre-existing common chronic medical conditions throughout the COVID-19 pandemic times, as well as the factors that contributed to them. The research will provide evidence to policymakers, program planners, and health care practitioners to help them make better decisions, as well as be valuable for evidence-based interventions in treatment of common mental health concerns during a future pandemic.

## Materials and methods

### Study design, area and period

We conducted a cross-sectional study at four hospitals in Sidama National Regional State, southern Ethiopia, between June 1 and September 1, 2021 [Hawassa university comprehensive specialized hospital (HUCSH), Adare general hospital (AGH), Yirgalem general hospital (YGH), and Leku Primary hospital (LPH)]. The Hawassa University comprehensive specialized hospital, with about 500 beds in southern Ethiopia, serves a population of over 18 million in the Sidama region and surrounding regions of southern Ethiopia, Oromia and Somalia. It is the only specialized hospital in the region. It was provided inpatient services for COVID-19 patients and isolation center in the region and surroundings. Yirgalem General Hospital is found in Yirgalem town and it is one of the General Hospitals in Sidama region serving about 4 million people in the region and nearby. Adare general hospital is located in Hawassa city and serves for communities from Hawassa and nearby cities. Leku primary hospital is one of the primary hospitals found in Sidama region located around 25 kilo meter away from Hawassa city.

### Study participants

In this study we have included patients with chronic NCDs such as diabetes, hypertension, chronic cardiovascular diseases, respiratory diseases such as asthma, and others who had regular follow-up visit at the selected hospitals. Individuals with pre-existing chronic medical illnesses receiving follow-up care in the outpatient departments (OPDs) of the four hospitals were contacted and requested to take part in the study if they met the following criteria: (I) patients above the age of 18 with confirmed chronic NCDs; (II) patients with stable clinical condition and able to understand the objective of the survey; and (III) patients with no known serious mental or neurocognitive problems. Patients with pre-existing chronic NCDs who were admitted to the emergency or inpatient units for any reason were not included in the study. The required sample size was estimated using single population proportion formula: (Z_α/2_)^2^ × p × (1 − p)/d^2^, where n is the sample size, z is the standard normal score set at 1.96, d is the desired degree of accuracy and p is the estimated proportion of the target population. By taking *p* = 50% (because there were no similar study in the area and to get adequate or maximum sample size), Zα/2 = 1.96 and d = 5%, the computed sample size was 384; and by taking 10% non-response rate, the total sample size computed was 422. Using design effect 1.5, our final sample size was estimated to be 633 (we used level of hospitals as a cluster, i.e., primary, general and specialized hospital). Sample size was proportionally allocated to each hospital based on the patient flow of the hospitals.

### Data collection methods

A standardized structured interviewer administered questionnaire was used to collect data. The questionnaire included items or scales that assess patients’ socio-demographic characteristics, clinical characteristics, social support, common mental disorders (CMD), insomnia, and quality of life (QOL). The questionnaire was written in English before being translated into Amharic, the local language. The questionnaire was translated from English into Amharic by native speakers of the language who are fluent in English, then back-translated into English by other translators to guarantee consistency. Finally, the Amharic version of the questionnaire was used to collect data.

Data were collected by nurses who had received 2 days of training on data collection processes and assessment tools. A pretest was performed on 5% of the sample to discover potential difficulties with the data collection procedures, as well as to assess the consistency of the questionnaires and the competency of the data collectors. The investigators reviewed each questionnaire for completeness on a regular basis during data collection.

### Data collection tools

#### Social support

Social support of patients with pre-existing chronic NCDs was measured using the three-item Oslo social support scale (OSSS). The scale has a score of minimum 3 and maximum 14. It has three categories. A score of 3–8, 9–11, and 12–14 indicates poor, moderate, and strong social support, respectively (41).

#### Common mental disorder

The World Health Organization developed the SRQ-20, a 20-item screening tool for common mental disorders (42). There are only binary (yes/no) questions, where “1” denotes the presence of a symptom and “0” denotes its absence. The SRQ-20 item questions categorize depression, anxiety, and psychosomatic issues as CMD (43). The SRQ-20’s validity, reliability, and cut-off differ according to the population (culture, language, setting, and gender) in various circumstances (43–46). The SRQ-20 exhibited good internal reliability (= 0.78) and an ideal cut-off score of 5/6, with a sensitivity of 78.6% and a specificity of 81.5% (47). The SRQ-20 measure demonstrated good internal consistency (Cronbach’s α = 0.89) in our study.

#### Insomnia

The nature, intensity, and effects of insomnia are evaluated using the ISI, a seven-item self-assessment questionnaire (48). The following elements are taken into account: the severity of difficulty of falling asleep, staying asleep, and waking up early in the morning; sleep dissatisfaction; how sleep issues affect daytime functioning; how others perceive the difficulty of sleep; and the distress caused by recent difficulty of sleep. Each item is rated on a 5-point Likert scale (0 = no problem; 4 = very severe difficulty, for example), with the total score ranging from 0 to 28. The overall score is divided into four categories: no insomnia (0–7), sub-threshold insomnia (8–14), moderate insomnia (15–21), and severe insomnia (22–28). A higher score illustrates severe insomnia (48–50). In the present study, the ISI measure showed very high internal consistency (Cronbach’s alpha = 0.96).

#### Quality of life

To analyze the impact of the COVID-19 pandemic on QOL, we used an adapted version of 12 items (51), from the WHOQOL-BREF scale (52, 53). The 12-item WHOQOL-BREF scale that was modified had a minimum score of 12 and a maximum score of 60. Due to the detrimental impacts of the COVID-19 pandemic, low scores indicate a lower QOL. In a prior study, the modified WHOQOL-BRIEF (12-items) showed acceptable internal consistency (Cronbach’s alpha = 0.81) (51). The 12-item WHOQOL-BRIEF that was used in our study had good internal consistency (Cronbach’s alpha = 0.82).

### Data analysis

For analysis, the data collected were entered into Epi-data version 3.1 and exported to SPSS version 24 for Windows. Various variables were described using descriptive statistics such as frequency, percentage, mean, standard deviation, and median. Binary and multivariable logistic regression were used to identify independent factors of insomnia and CMD. The Hosmer-Lemeshow test was used to assess model fitness. At 95% confidence interval (CI), *p*-values of <0.05 were considered as statistically significant.

## Results

### Socio-demographic characteristics of study participants

A total of 633 patients with pre-existing chronic NCDs were involved in this study. Majority were male 357(56.4%), 410(64.8%) participants were married, 352(55.6%) were Protestant by religion and about one-fourth 175(27.6%) were illiterate. Of the total participants, about one-fifth 144(22.7%) were farmer by occupation and majority 353(55.8%) were urban residents. The mean age of the respondents was 46.49 ± 17.71 as described in Table 1.

**Table 1:** Socio-demographic characteristics of study participants at Sidama National Regional State, southern Ethiopia, 2021 (*n* = 633).

Variable	Categories	Frequency	Percentage (%)
Age (mean ± SD)	46.49 ± 17.71		
Sex	Male	357	56.4
	Female	276	43.6
Marital status	Single	135	21.3
	Married	410	64.8
	Divorced	36	5.7
	Widowed	52	8.2
Religion	Protestant	352	55.6
	Orthodox	164	25.9
	Muslim	103	16.3
	Others	14	2.2
Educational status	Illiterate	175	27.6
	Primary	162	25.6
	Secondary	131	20.7
	College and above	165	26.1
Occupation	Gov’t employee	121	19.1
	Private employee	67	10.6
	Merchant	90	14.2
	Student	68	10.7
	House wife	102	16.1
	Farmer	144	22.7
	Jobless	21	3.3
	Other	20	3.2
Place of residence	Rural	280	44.2
	Urban	353	55.8

### Clinical characteristics of study participants

About half 336(53.1%) of the participants were diagnosed with diabetes mellitus followed by hypertension 111(17.5%) and nearly one-third 200(31.6%) participants had comorbid diagnosis. Majority 364(57.5%) of the participants were ill for ≤5 years, 61(9.6%) and 49(7.7%) participants were using alcohol and khat in the past three months, respectively. Among the total participants, more than one-third 224(35.4%) and about half 332(52.4%) had poor and moderate social support, respectively. Moreover, 293(46.3%) of participants were poor QOL (Table 2).

**Table 2:** Clinical characteristics of study participants at Sidama National Regional State, southern Ethiopia, 2021 (*n* = 633).

Variable	Categories	Frequency	Percentage (%)
Diagnosis	Diabetes Mellitus	336	53.1
	Hypertension	111	17.5
	Asthma	49	7.7
	CVD	82	13.0
	Others*	55	8.7
Comorbid diagnosis	Yes	200	31.6
	No	433	68.4
Duration of illness	<= 5 years	364	57.5
	6–10 years	210	33.2
	> = 11 years	59	9.3
Alcohol Use in the past 3 months	Yes	61	9.6
	No	572	90.4
Cigarette Use in the past 3 months	Yes	17	2.7
	No	616	97.3
Khat Use in the past 3 months	Yes	49	7.7
	No	584	92.3
Social support	Poor	224	35.4
	Moderate	332	52.4
	Strong	77	12.2
Quality of life	Good	340	53.7
	Poor	293	46.3

CVD, Cardio-vascular disorders, *Epilepsy, stroke, neurological, renal or hepatologic disorders.

### Prevalence of insomnia and CMD

**Figure 1:**
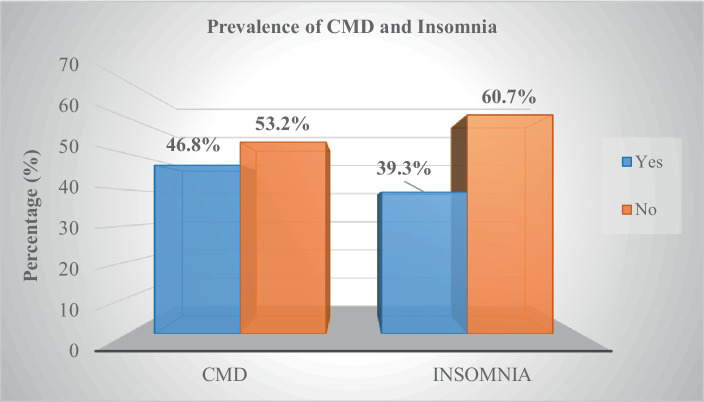
Prevalence of insomnia and CMD study participants at Sidama National Regional State, southern Ethiopia, 2021 (*n* = 633).

The prevalence of insomnia and CMD were found to be 39.3% (95% CI = 35.3–43.3%) and 46.8% (95% CI = 42.8–50.6%), respectively as shown in Figure 1.

### Independent predictors of CMD and insomnia

In a multivariable logistic regression model, occupation, diagnosis, comorbid diagnosis, social support, insomnia, and quality of life were all significantly associated with CMD. When compared to government employees, being a merchant is 66% (AOR = 0.33; 95% CI = 0.13, 0.82) less likely to develop CMD. Regression analyses revealed that having a diagnosis of diabetes mellitus (AOR = 1.89; 95% CI = 1.04, 3.46) and a comorbid diagnosis (AOR = 3.96; 95% CI = 2.27, 6.89) increased the likelihood of reporting CMD significantly more than having a diagnosis of hypertension and their counterparts. Poor social support (AOR = 3.37; 95% CI = 1.51, 7.57) and moderate social support (AOR = 3.13; 95% CI = 1.46, 6.69) were both associated with an increased risk of developing CMD. Individuals with symptoms of insomnia (AOR = 12.08; 95% CI = 7.41, 19.72) and poor QoL (AOR = 1.67; 95% CI = 1.04, 2.72) were more likely to report CMD as shown in Table 3. We also found out that, having CVDs (AOR = 2.48; 95% CI = 1.18, 5.19), CMD (AOR = 12.09; 95% CI = 7.46, 19.61), and poor QOL (AOR = 2.04; 95% CI = 1.27, 3.26) increased the odds of having insomnia (Table 4).

**Table 3:** Factors associated with CMD among patients with chronic NCDs during COVID-19 pandemic in selected hospitals of Sidama national regional state, 2021 (*n* = 633).

*Variable*	*Category*	*CMD*		*COR (95% CI)*	*AOR (95% CI)*
		Yes	No		
Age	≤ 30 years	22	115	1	1
	31–45 years	63	133	2.48(1.44, 4.27) *	1.19(0.47, 3.01)
	46–60 years	58	120	2.53(1.45, 4.39) *	1.41(0.52, 3.77)
	≥ 61 years	66	56	6.16(3.45, 10.99) *	1.03(0.33, 3.21)
Marital status	Single	31	104	1	1
	Married	125	285	1.47(0.94, 3.31) **	0.60(0.26, 1.37)
	Divorced	23	13	5.94(2.69, 13.07) *	1.91(0.49, 7.33)
	Widowed	30	22	4.57(2.32, 9.04) *	0.64(0.19, 2.14)
Educational status	Illiterate	57	118	0.69(0.44, 1.07) **	1.67(0.71, 3.95)
	Primary	44	118	0.53(0.33, 0.85) *	1.18(0.53, 2.64)
	Secondary	40	91	0.63(0.38, 1.02) **	1.02(0.45, 2.29)
	Tertiary	68	97	1	1
Occupation	Gov’t employee	68	53	1	1
	Private employee	41	26	1.23(0.67, 2.25)	1.22(0.51, 2.95)
	Merchant	29	61	0.37(0.21, 0.65) **	0.33(0.13, 0.82)*
	Student	13	55	0.18(0.09, 0.37) ***	0.46(0.14, 1.58)
	House wife	53	49	0.84(0.49, 1.43)	0.86(0.33, 2.21)
	Farmer	71	73	0.75(0.46, 1.23)	0.63(0.25, 1.62)
	Jobless	7	14	0.39(0.14, 1.03)	0.57(0.14, 2.41)
	Other	14	6	1.82(0.65, 5.05)	1.14(0.25, 5.10)
Diagnosis	Hypertension	107	229	1	1
	Diabetes Mellitus	32	79	1.43(0.93, 2.21)	1.89(1.04, 3.46)*
	Asthma	22	27	2.13(1.07, 4.22) *	1.04(0.41, 2.66)
	CVD	40	42	1.87(1.05, 3.34) *	1.18(0.54, 2.61)
	Others	8	47	0.33(0.15, 0.71) **	0.23(0.08, 0.63)**
Duration of illness	≤ 5 years	101	263	1	1
	6–10 years	83	127	1.70(1.19, 2.44) *	1.02(0.62, 1.67)
	≥ 11 years	25	34	1.92 (1.09, 3.37) *	1.28(0.60, 2.75)
Comorbid diagnosis	Yes	119	81	5.60(3.89, 8.07) *	3.96(2.27, 6.89) ***
	No	90	343	1	1
Family History of MI	Yes	18	11	3.54(1.64, 7.64) *	2.65(0.85, 8.25)
	No	191	413	1	1
Alcohol Use in the past 3 months	Yes	38	23	3.87(2.24, 6.70) *	1.47(0.55, 3.94)
	No	171	401	1	1
Cigarette Use in the past 3 months	Yes	11	6	3.87(1.41, 10.62) *	0.80(0.13, 5.04)
	No	198	418	1	1
Khat Use in the past 3 months	Yes	29	20	3.25(1.79, 5.91) *	2.74(0.91, 8.29)
	No	180	404	1	1
Social support	Poor	64	160	1.31(0.72, 2.39)	3.37(1.51, 7.57) **
	Moderate	127	205	2.03(1.14, 3.60) *	3.13(1.46, 6.69) **
	Strong	18	59	1	1
Insomnia	Yes	164	85	14.54(9.68, 21.83) *	12.08 (7.41, 19.72) ***
	No	45	339	1	1
Quality of life	Good	123	217	1	1
	Poor	173	120	3.11(2.23, 4.33)	1.67(1.04, 2.72) *

NB: *p < 0.05, **p < 0.01, ***p < 0.001; CVD, Cardio-vascular disorders; CMD, Common Mental Disorder.

**Table 4:** Factors associated with Insomnia among patients with chronic NCDs during COVID-19 pandemic in selected hospitals of Sidama national regional state, 2021 (*n* = 633).

Variable	Category	Insomnia		COR (95% CI)	AOR (95% CI)
		Yes	No		
Age	≤ 30 years	30	107	1	1
	31–45 years	75	121	2.21(1.35, 3.63) *	1.06(0.43, 2.63)
	46–60 years	71	107	2.37(1.43, 3.92) *	0.93(0.35, 2.44)
	≥ 61 years	73	49	5.31(3.09, 3.15) *	1.36(0.46, 4.02)
Marital status	Single	34	101	1	1
	Married	157	253	1.84(1.19, 2.85) *	1.05(0.46, 2.37)
	Divorced	22	14	4.67(2.15, 10.13) *	1.22(0.39, 3.79)
	Widowed	36	16	6.68(3.30, 13.54) *	2.54(0.78, 8.20)
Educational status	Illiterate	74	101	0.90(0.58, 1.38)	0.94(0.41, 2.18)
	Primary	54	108	0.62(0.39, 0.96) *	0.92(0.43, 1.96)
	Secondary	47	84	0.68(0.43, 1.10)	1.16(0.54, 2.50)
	Tertiary	74	91	1	1
Occupation	Gov’t employee	57	64	1	1
	Private employee	32	35	1.03(0.56, 1.86)	0.69(0.32, 1.52)
	Merchant	27	63	0.48(0.27, 0.85) *	0.75(0.31, 1.83)
	Student	10	58	0.19(0.09, 0.41) **	0.46(0.13, 1.61)
	House wife	49	53	1.04(0.61, 1.75)	1.25(0.49, 3.15)
	Farmer	58	86	0.75(0.46, 1.23)	0.71(0.28, 1.82)
	Jobless	6	15	0.45(0.16, 1.23)	0.64(0.17, 2.36)
	Other	10	10	1.12(0.44, 2.89)	0.61(0.15, 2.42)
Diagnosis	Hypertension	112	224	1	1
	Diabetes	39	72	0.92(0.58, 1.45)	0.63(0.34, 1.13)
	Asthma	27	22	2.26(1.14, 4.49) *	1.95(0.81, 4.69)
	CVD	47	35	2.47(1.38, 4.45) **	2.48(1.18, 5.19)*
	Others	24	31	1.43(0.74, 2.76)	3.38(1.45, 7.88)**
Duration of illness	≤ 5 years	128	236	1	1
	6–10 years	96	114	1.55(1.09, 2.19) *	1.21(0.74, 1.95)
	≥ 11 years	25	34	1.36(0.77, 2.37)	0.68(0.31, 1.52)
Comorbid diagnosis	Yes	109	91	2.51(1.78, 3.53) *	0.87(0.52, 1.49)
	No	140	293	1	1
Alcohol Use in the past 3 months	Yes	43	18	4.24(2.38, 7.55) *	1.82(0.80, 4.13)
	No	206	366	1	1
Cigarette Use in the past 3 months	Yes	13	4	5.23(1.68, 16.24) *	1.59(0.29, 8.65)
	No	236	380	1	1
Khat Use in the past 3 months	Yes	35	14	4.32(2.27, 8.23) *	2.43(0.93, 6.30)
	No	214	370	1	1
CMD	Yes	198	98	1	12.09(7.46, 19.61) ***
	No	51	286	11.33(7.72, 16.63) **	1
Quality of life	Good	92	248	1	1
	Poor	157	136	3.11(2.23, 4.33) **	2.04(1.27, 3.26) **

NB: *p < 0.05, **p < 0.01, ***P < 0.001; CVD, Cardio-vascular disorders; CMD, Common Mental Disorder.

## Discussion

The COVID-19 pandemic appears to be having a severe impact on the mental health of individuals with pre-existing medical conditions. In this study, we examined the prevalence and predictors of COVID-19 pandemic related common mental disorders (CMD) and insomnia in patients with chronic medical conditions in south Ethiopia. CMD and insomnia levels were surprisingly high, according to our research. Being a merchant, having diabetes mellitus, having a longer illness duration, having comorbid diagnoses, having low social support, having comorbid insomnia, and having a poor quality of life (QoL) were significant predictors of CMD. Additionally, a significant association between a high risk of insomnia and a diagnosis of cardio-vascular disorders, concurrent CMD, and poor QoL was found. The prevalence of insomnia and CMD indicates a need for mental health and psychosocial support (MHPSS) during the COVID-19 pandemic.

CMDs such as anxiety and depression were found to be more common in pre-existing physical conditions with a higher risk of COVID-19 infection in previous studies (54, 55). Our finding suggests that nearly half (46.8%) of the participants experience CMD. Comparable findings were reported from previous studies in India (52.9%), Saudi Arabia (45.6%), and China (45%) (56–58). This shows that COVID-19 had a serious impact on the mental health of people with pre-existing NCDs. Mental health of the population was significantly impacted by the public health measures implemented to stop the spread of COVID-19 and the fear of getting the infection (59). The association between chronic medical problems and mental disorders is generally speculated to be bidirectional. People suffering from a significant mental illness are more likely to develop a variety of chronic physical illnesses. People with chronic medical illnesses, on the other hand, more likely to experience psychological problems than the general population (60). For instance, compliance with chronic medication use and regular use of the healthcare system may be challenging during infectious disease outbreaks, adding to psychological distress.

However, lower prevalence were reported in Ethiopia (22.8%) (61), and India (33.2%) (62). A similar study conducted in Bangladesh found a low prevalence of anxiety (35.2%) and depression (38.9%) (60). On the other hand, higher prevalence were also reported in studies conducted in Harar (62%) (60), and Mettu Karl Referral Hospital 55.7% (depression) and 61.8% (anxiety) (59). Moreover, other pre-COVID-19 researches indicated higher estimates of mental health problems in different parts of the world such as 63.3% in Ethiopia (63), 60.2% in Pakistan (64), 64.4% in Nigeria (65), and (58.7%) in Delhi, India (66). These disparities could be due to changes in sample size, inclusion criteria, screening instrument, setting, study period, and study population. Consequently, physical illnesses were associated with a higher likelihood of mental health problems, which could be explained by underlying physical disorders and a lack of medical help during an epidemic (67, 68). The findings suggested that different strategies should be designed to address mental health issues during epidemics.

This result of this study revealed that the prevalence of insomnia among chronic medical conditions was found to be 39.3%. Previous investigations and systematic review reports in different situations and populations provided almost approximate results in line with our findings (69–72). Similar results were recently observed in a variety of populations, including outpatients (39.5%), healthcare professionals (44.6%), and general population (39.8%) (73) and patients with chronic medical illness (32.6%) (74), during the COVID-19 pandemic. However, lower prevalence were also reported by Nochaiwong et al., (28%) and Liu et al., (30%) among general population and Mulyadi et al., (33%) among college students (75–77). On the other hand, higher prevalence were reported in COVID-19 patients (57%), cancer outpatients (48.6%), and depression outpatients (80.9%), individuals living with disabilities (71%) during the COVID-19 era (78–81). Therefore, variations in people’s underlying medical conditions may account for variations in the prevalence rates of insomnia.

Being a governmental employee increases the likelihood to have CMD as compared to merchants. This finding is consistent with a recent study (82). The civil servants who participated in COVID-19 control were reported to have various types of psychological disorders (83). It has been noted that lack of public support, long working hours, a high degree of work related stress, work instability, and an unfavorable physical environment are risk factors for the development of symptoms of mental disorders in government employees (84–86). Furthermore, many government employees encounter emotional distress due to psychological disorders like depression and anxiety during and even after pandemics (87).

Similarly, those with inadequate (poor and moderate) social support were more likely to have CMD than those with strong social support, which is consistent with previous studies (59, 88, 89). Individuals with higher levels of social support may be more likely to believe they will obtain the necessary assistance when confronted with the stressful event of a pandemic outbreak. This idea would improve their views about dealing with adversity and difficulty in the fight against COVID19, resulting in greater levels of resilience (88). This could be associated with the fact that those who did not have social support throughout the pandemic are more vulnerable to mental illness since their social health is disrupted (61). Furthermore, a lack of social support has a detrimental effect on self-care, adherence, and the ability to react to or deal with stressful events, and it may lead to the development of depression (90). Also, perceived social support may help individuals resist and successfully cope the risk factors associated with their mental health (91). As a result, our findings highlight the importance of strong social support during pandemics, as it may have a positive impact on mental health.

This study indicated that patients diagnosed with diabetes were more prone to develop CMD as compared to hypertension. Mental health problems such as depression is more likely to occur in people with diabetes (92–94). Several studies have recently revealed that patients with diabetes mellitus had significant psychological problems during the COVID-19 pandemic (95–98). This has been linked to hypothalamic–pituitary–adrenal (HPA) dysfunction in both diabetes and depression (99). The immune system, glucose metabolism, and sleep—indicators of impaired health in both diseases—are regulated by this route, which is crucial under stress (100). Moreover, we found out that patients diagnosed with cardiovascular disorders such as heart failure were more likely to develop insomnia as compared to patients with hypertension. Previous studies suggested that patients with CVD are more prone to sleep disturbances (101, 102). Insomnia is linked to fatigue, depressed symptoms, daytime sleepiness, and decreases in self-reported and objective functional status, all of which are major issues for CVD patients (103). This could be explained by the hypothalamic–pituitary axis (HPA) dysregulation, inappropriate modulation of the autonomic nervous system, elevated sympathetic nervous system activity, and systemic inflammation that are all factors in the etiology of cardiovascular disease (102). In addition, several pandemic-specific factors, such as fear of acquiring the virus and being unable to visit loved ones, suggest that insomnia may increase during this time (104).

In this study, participants with CMD were more likely to be at risk to develop insomnia and vice versa. In general, there is evidence that mental health problems are associated to sleep disturbances and that insomnia is related to the psychological stress. That is, a bidirectional relationship between insomnia and psychological problems were clearly demonstrated (105). Furthermore, people who have a chronic medical condition may experience severe physical discomfort, which can lead to anxiety and depression and disturbed sleep (106). The conclusion could be attributed to COVID-19’s direct psychosocial effects on those with chronic medical conditions, fear of contracting COVID-19, and the virus’s deadly effect on chronic medical patients.

Our study also showed that participants who had poor QoL were more likely to develop CMD and insomnia. Similarly, recent studies have discovered that poor QoL is associated with insomnia and poor sleep quality (107, 108) and lower QoL scores were indicated by individuals who felt more stress, depression, and anxiety (51). Previous research has shown that there is an inverse relationship between QOL and mental health disorders such as depression and anxiety (109) and sleep disturbances (110). As a result, it is not surprising that poor QOL in individuals with chronic medical illnesses has an impact on mental health and sleep, as shown in this study.

There were certain limitations in this study. First, due to the study’s cross-sectional methodology, it was unable to demonstrate causal links between the variables investigated. Second, because all of the data was self-reported and the participants’ responses were not independently validated, social desirability bias and recall bias could have occurred. Third, because the study’s participants were all people with chronic illnesses, the findings cannot be generalized to the general public.

## Conclusion

This study highlights the prevalence of CMD and Insomnia among patients with chronic NCDs were found to be considerably high. Occupation, diagnosis, comorbid diagnosis, social support, insomnia, and quality of life were all significantly associated with CMD. We also found out that, having CVDs, CMD, and poor QOL significantly increases the likelihood of having insomnia symptoms. As a result, addressing mental health issues among patients with chronic CMD is an essential component of public health interventions during the COVID-19 pandemic.

## Data availability statement

The original contributions presented in the study are included in the article/supplementary material, further inquiries can be directed to the corresponding author.

## Ethics statement

The studies involving human participants were reviewed and approved by Hawassa University, College of Medicine and Health Sciences, Institutional Review Board (IRB) with reference number IRB/076/13. The participants provided their written informed consent to participate in this study.

## Author contributions

MA, BD, AG, SH, and ET participated in the conception and designed the study and involved in the data collection. MA, BD, and SD do the analysis of the study. MA and SD prepares the manuscript for publication. BD, AG, SH, SD, and ET critically reviewed the manuscript. All authors contributed to the article and approved the submitted version.
